# Hydrothermal synthesis of cobalt telluride nanorods for a high performance hybrid asymmetric supercapacitor

**DOI:** 10.1039/c9ra08692g

**Published:** 2020-04-03

**Authors:** M. Manikandan, K. Subramani, M. Sathish, S. Dhanuskodi

**Affiliations:** School of Physics, Bharathidasan University Tiruchirappalli – 620 024 Tamil Nadu India dhanus2k3@yahoo.com; Electrochemical Power Sources Division, CSIR-CECRI Karaikudi – 630 003 Tamil Nadu India msathish@cecri.res.in; Academy of Scientific and Innovative Research (AcSIR) Ghaziabad-201002 India

## Abstract

Cobalt telluride nanostructured materials have demonstrated various applications, particularly in energy generation and storage. A high temperature and reducing atmosphere are required for the preparation of cobalt telluride-based materials, which makes this a difficult and expensive process. The development of a facile route for producing the desirable nanostructure of cobalt telluride remains a great challenge. We demonstrated a simple hydrothermal method for preparing cobalt telluride nanorods (CoTe NRs) and telluride nanorods (Te NRs) for supercapacitor applications. The morphology of CoTe NRs and Te NRs was analyzed using scanning electron microscopy (SEM) and transmission electron microscopy (TEM). The prepared CoTe NR electrode material exhibited a high specific capacity of 170 C g^−1^ at a current density of 0.5 A g^−1^ with an exceptional cyclic stability. The asymmetric supercapacitor was assembled using CoTe NRs and orange peel-derived activated carbon (OPAA-700) as a positive and negative electrode, respectively. The fabricated device delivered a high energy density of 40.7 W h kg^−1^ with a power density of 800 W kg^−1^ at 1 A g^−1^ current density. When the current density was increased to 30 A g^−1^, the fabricated device delivered a high power density of 22.5 kW kg^−1^ with an energy density of 16.3 W h kg^−1^. The fabricated asymmetric supercapacitor displayed a good cyclic stability performance for 10 000 cycles at a high current density of 30 A g^−1^ and retained 85% of its initial capacity for after 10 000 cycles. The prepared materials indicate their applicability for high performance energy storage devices.

## Abstract 2

A one-step hydrothermal derived cobalt telluride nanorods and activated carbon-based hybrid asymmetric supercapacitor delivered a high energy (40.7 W h kg^−1^) and power density (22.5 kW kg^−1^) with an electrochemical stability of 85% for 10000 cycles.

## Graphical abstract

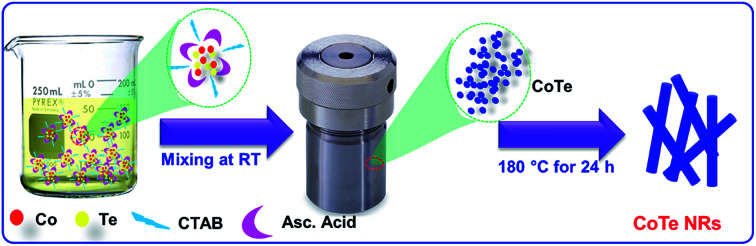

## 1. Introduction

In 21^st^ century, the global demand in the energy sectors and limited availability of fossil fuels require society to move towards substantial developments in the energy conversion and storage devices.^1,2^ For energy storage, batteries and conventional capacitors are the main devices that can deliver a high energy and high power, respectively. Currently, batteries possess a high energy density and low power density, while the conventional capacitor delivers a high power but limits its energy storage.^3,4^ Supercapacitors, also known as ultra-capacitors, have drawn a great interest in recent years because of their high power performance, fast charge/discharge ability, long cycle life and low cost.^5–11^ Commercial-based symmetric supercapacitors in non-aqueous electrolytes have delivered high-power densities in the range of 12–14 kW kg^−1^ with energy densities of 4–6 W h kg^−1^. So, the progress of supercapacitors continues mostly towards the development of high energy and low-cost nanostructured electrode materials. The energy density of the supercapacitor depends on the capacity of the material and cell voltage. However, the low cell potential of the aqueous-based symmetric supercapacitor limits its potential application due to water decomposition. Recently, hybrid asymmetric supercapacitors in an aqueous electrolytes have been developed in which the faradaic metal oxide/hydroxide/sulfide/telluride and an EDLC-based carbon electrode materials are used as the positive and negative electrodes, respectively.

Hybrid supercapacitors can efficiently utilize the potential gap between the two types of electrodes to increase the cell voltage. The energy density of the supercapacitor materials depends on the capacity of the electrode material and cell voltage, while increasing the cell voltage the energy density get enhanced. Ni-, Co- and Fe-based battery-type electrode materials are promising materials for high energy storage applications due to the low cost, environmental sustainability, intrinsic electronic conductivity, high capacity, good rate performance, long life span and high redox behavior.^12–16^ For example, Wang *et al.* fabricated a Ni_3_S_4_ battery-type electrode supercapacitor with a specific capacitance of 898.5 C g^−1^ at 0.5 A g^−1^ current density.^17^ Ni_3_S_2_/CdS exhibit a capacity of 545.6 C g^−1^ at the current density of 1 A g^−1^ with the capacitance retention of 103% after 5000 cycles.^18^ Liu *et al.* reported on Ni–Co@Ni–Co layered double hydroxide for solid state battery-type supercapacitor. It gives an energy density of 100 W h kg^−1^ and cyclic stability of 98.6%.^19^ Ce-doped α-Co(OH)_2_ shows a specific capacity of 415 C g^−1^ with a cyclic stability of 73%, even after 2000 cycles.^20^

To obtain a new device with a large energy density, power density and potential window of the asymmetric supercapacitor (ASC) has received a great attention by the combination of two different types of electrodes to make asymmetric configurations resulting in the storage of more energy and a wider cell potential due to the utilization of faradaic and non-faradaic process in a single device. In ASC, the faradaic electrode as the positive electrode (energy source) generally uses transition metals such as Ni(OH)_2_ and CoO because of their high specific capacity and electrochemical performance. The EDLC as a negative electrode (power source) uses materials like activated carbon, carbon nanotubes and graphene because of their high surface area, high stability and high electrical conductivity.^21,22^ In this scenario, metal chalcogenides are a new type of electrode material for the electrochemical supercapacitor. In the metal chalcogenides, the metal ion undergoes fast surface redox reactions associated with different oxidations of the transition metal ion. Transition metal chalcogenides like NiS, CoS, CuS, MoS_2_, NiSe, CoSe, and MoSe_2_ nanostructures are more interesting towards supercapacitor applications due to the high surface area, specific capacitance, excellent electrochemical properties, high electrical conductivity and stability.^23^

Recently, metal tellurides like CoTe, NiTe, La_2_Te_3_, MoTe_2_ and Sm_2_Te_3_ have been used as an electrode materials for supercapacitor applications. Among these metal tellurides, CoTe is of immense interest due its high theoretical capacity of 1034 C g^−1^. CoTe nanowires can extend their potential voltage to 1.6 V and give energy and power density values of 32.9 W h kg^−1^ and 800.27 W kg^−1^, respectively, even after 5000 cycles at the current density (5 A g^−1^) with an initial capacitance of 90.5%, as reported by Xiao *et al.*^21^ Ye *et al.* synthesized a CoTe‖AC asymmetric supercapacitor electrode grown on carbon fiber paper by hydrothermal method, which showed a specific capacitance of 120 C g^−1^ with an energy density of 67.0 W h kg^−1^.^22^ Cao *et al.* reported on Te NWs arrays with high conductivity grown on a flexible carbon fiber to fabricate hybrid Te/Au/MnO_2_ core–shell nanostructures as a supercapacitor with 97% capacitance retention for 1000 cycles.^24^ Zhou *et al.* prepared NiTe rods as a positive electrode material for an asymmetric supercapacitor (ASC) with a specific capacitance of 804 F g^−1^ (current density 5 A g^−1^) and 91% of capacitance retention for 1000 cycles.^25^ Liu *et al.* recorded that few-layer 1T′ MoTe_2_ obtained a high specific capacitance of 835 C g^−1^ at a current density of 1 A g^−1^.^26^ Kumbhar *et al.* synthesized Sm_2_Te_3_ thin films through a one-step chemical route with a specific capacitance of 129 C g^−1^ with high energy and power density values of 25.60 W h kg^−1^ and 14.18 kW kg^−1^, respectively.^27^ Patil *et al.* successfully synthesized La_2_Te_3_ thin film *via* a successive ionic layer adsorption and reactive method for supercapacitor charge storage properties. It exhibited a specific capacitance of 161 C g^−1^ with a capacitance retention of 82% over 1000 cycles at a scan rate of 100 mV s^−1^.^28^ In our previous work, we demonstrated a nickel telluride nanorod (NiTe NR) by hydrothermal method using ascorbic acid and CTAB as the reducing agent and surfactant, respectively. High specific capacitances of 370 C g^−1^ were achieved at the current density of 1 A g^−1^ with good electrochemical cyclic stability.^29^ Here, all capacitance values are recalculated as capacity values for consistency.

CoTe has attracted great interest because its thermal, electrical and magnetic properties have shown a variety of applications such as electrocatalysis,^30^ water splitting,^31^ solar cells,^32^ batteries,^33^ photocatalysis^34^ and biosensors.^35^ Cobalt telluride nanostructure materials have demonstrated various applications particularly in energy generation and storage. High temperatures and a reducing atmosphere are needed for the preparation of cobalt telluride-based 2-D nanostructure materials, which makes it difficult and expensive for the large-scale production and fabrication of supercapacitors. Thus, the development of a facile route for desirable CoTe nanostructures remains a great challenge. To the best of our knowledge, this is the first report on hydrothermally prepared CoTe and Te NRs as electrode materials for supercapacitor applications. The as-prepared CoTe NR electrode material delivered a high specific capacity of 170 C g^−1^ at a current density of 0.5 A g^−1^, which is much higher than the Te NRs. Depending on the growth condition of the materials, it can exhibit various surface morphologies. The capacity of the materials differs from the different morphologies because of both faradaic and nonfaradaic processes. Here, the CoTe surface area is higher than Te. In the case of morphology, it is the opposite. Asymmetric supercapacitors (ASC) were fabricated using CoTe NRs and orange peel-derived activated carbon (OPAA)^36^ as positive and negative electrodes, respectively. The fabricated device delivered a high energy density and power density of 40.7 W h kg^−1^ and 22.5 kW kg^−1^, respectively. In addition, the fabricated device exhibited an excellent rate performance and good cycling stability, which make it a potential electrode material for energy storage applications.

## 2. Experimental

### 2.1. Preparation of CoTe nanorods

Sodium telluride (Na_2_TeO_3_, Sigma Aldrich), cobalt acetate (Co(CH_3_COO)_2_·4H_2_O, Sigma Aldrich), ascorbic acid (C_6_H_8_O_6_), cetyltrimethylammonium bromide (CTAB, Sigma Aldrich) and deionized water from a Milli-Q-ultra pure water system (18.2 MΩ cm^−1^) were used as the starting materials. In the hydrothermal method, 14.2 mmol of C_6_H_8_O_6_ was dissolved in 40 ml of deionized water. Then, 0.82 mmol of CTAB was added gradually and stirred for 30 min. In the next step, 1.88 mmol of Na_2_TeO_3_ and 1.88 mmol of Co(CH_3_COO)_2_·4H_2_O was dissolved in 30 ml of deionized water and slowly added to the above solution and a white TeO_2_ precipitate was obtained immediately. Finally, the resultant solution was transferred into a 100 ml Teflon-lined stainless-steel autoclave that was sealed and maintained at 180 °C for 24 h in an oven, then allowed to cool to room temperature. The obtained black powder was collected and washed with deionized water and ethanol several times, and dried at room temperature. For comparison, pure Te was prepared in the absence of cobalt acetate by the same method.^37^

### 2.2. Preparation of orange peel-derived activated carbon

For the full cell fabrication of ASC, OPAA was used as a negative electrode material. The detailed synthesis procedure and characterizations are described in our previous publications.^36^ In detail, the dry orange peel powder was subjected to carbonization at 600 °C for 3 h in an argon (Ar) atmosphere and followed by chemical activation. The carbonized sample was mixed with KOH in a weight ratio of 1 : 3, and the obtained slurry was again heat-treated at 700 °C for 1 h in an Ar atmosphere to obtain the chemically activated carbon. The resulting activated carbon was washed thoroughly with 1 M HCl solution, followed by DI water until the pH of the sample became neutral. The resulting activated carbon was dried at 80 °C for 5 h.

### 2.3. Characterizations

The phase formation and crystalline nature of the synthesized materials were analyzed by powder X-ray diffraction (XRD) analysis using PANalytical XRD X'pert pro with Cu Kα radiation (*α* = 1.5418 Å). The elemental composition and oxidation states of the metal ions were examined by X-ray photoelectron spectroscopy (XPS; MULTILAB 2000, Thermo Scientific) using Mg Kα (1253.6 eV) as the X-ray source. The surface morphology and particle size of the synthesized materials have been studied by scanning electron microscope (SEM), Hitachi S-3000 H and transmission electron microscope (TEM), Jeol Jem-2100, respectively. An N_2_ adsorption–desorption experiment was carried out using a Micromeritics ASAP 2020 analyzer, and the surface areas were determined using BET (Brunauer–Emmett–Teller). The electrochemical performance of the electrode materials for supercapacitor application was evaluated by cyclic voltammetry (CV), galvanostatic charge discharge (GCD) and electrochemical impedance spectroscopy (EIS) analysis using electrochemical workstation (Biologic- SP300).

### 2.4. Preparation of electrode for supercapacitor

The working electrode was fabricated by mixing the active materials, acetylene black and polyvinylidene fluoride (PVDF) with a weight ratio of 75 : 20 : 5, respectively. This mixture was continuously ground in a mortar and 0.5 ml *N*-methyl-2-pyrrolidone (NMP) was used as a solvent to make a homogenous slurry. This homogenous slurry was coated on a nickel foam (2 × 2 cm^2^) current collector, and then dried at 90 °C for 12 h in a vacuum oven. The weight of the active material (∼4 mg) was optimized based on the weight of the Ni foam before and after coating. In a three-electrode configuration, active material-coated Ni foam, Pt foil and Hg/HgO (20% KOH) served as the working, counter and reference electrodes, respectively. The CV and GCD measurements were carried out using 3.5 M KOH as the electrolyte with different scan rates from 5 to 100 mV s^−1^ and different current densities from 1 to 10 A g^−1^, respectively. The electrochemical impedance spectroscopic (EIS) study was also carried out by applying an alternate current (AC) with a bias voltage of 5 mV amplitude in the frequency range from 100 kHz to 10 mHz. The specific capacity of the electrode materials was calculated from the following formula:^4^1
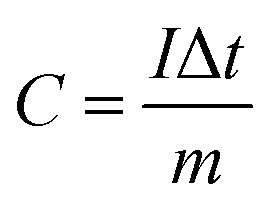
where *C* is the specific capacity of the electrode materials (C g^−1^), *I* is the discharge current (A g^−1^), Δ*t* is the discharge time (s), and *m* is the mass of the active material (g). The theoretical capacity of the electrode was calculated using the equation:2
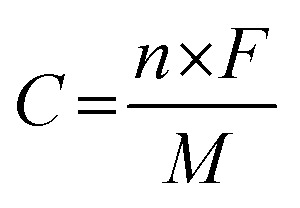
where *n* is the number of electrons transferred in the redox reaction, *F* is the Faraday constant, *M* is the molar mass and *V* is the operating potential window.

The hybrid asymmetric supercapacitor (ASC) was constructed using the faradaic type CoTe NRs as a positive electrode, EDLC-type activated carbon derived from orange peel biomass-derived activated carbon as a negative electrode with 3.5 M KOH as an electrolyte. The advantages of the ASC are a higher cell potential with a high specific capacity. To obtain better ASC performance, it is necessary to maintain the charge balance between the positive and negative electrodes using the following relationship: *q*_+_ = *q*_−_, where *q*_+_ and *q*_−_ are the stored charges in the positive and negative electrode surfaces, respectively. The amount of charge stored on the electrodes depends on the capacity (*C*) and mass of the electrode materials (*m*). Thus, the mass balance equation is expressed as follows:^3,38^

3 *q*_+_ = *C*_+_ × *m*_+_

4 *q*_−_ = *C*_−_ × *m*_−_

5
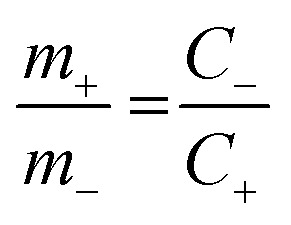
where *C*_+_ and *C*_−_ are the capacity of the positive and negative electrodes, and *m*_+_ and *m*_−_ are the mass of the positive (4.66 mg) and negative electrodes (2 mg). The capacity of the OPAA material (specific capacity is 280 C g^−1^ at 2 A g^−1^ current density) was calculated based on our previous work.^14^ The mass ratio for optimal electrochemical performance of ASC between the positive and negative electrodes was calculated to be 2.33. The energy and power density values were calculated by the following equations:^39^6
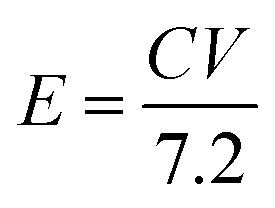
7
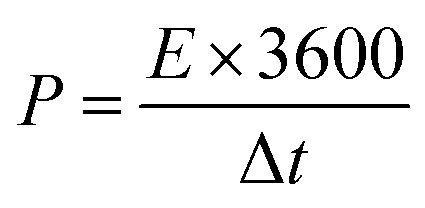
where the energy density (*E*), power density (*P*), *C* is the specific capacitance (c g^−1^), Δ*V* is the potential window (V), and Δ*t* is the discharging time (s).

## 3. Results and discussion

**Scheme 1:**
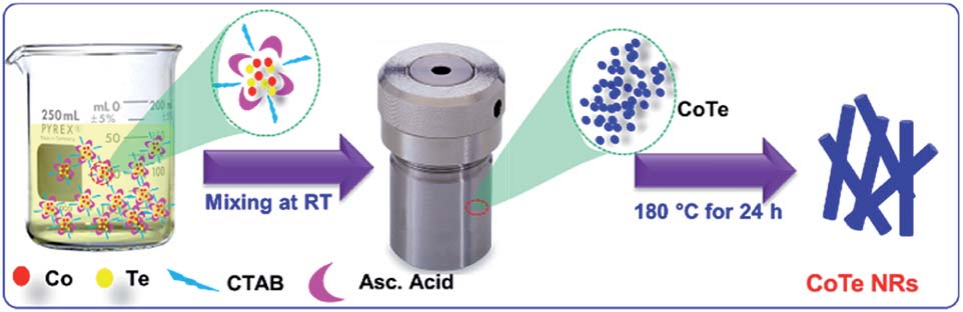
Schematic representation for the growth of CoTe nanorods.

Scheme 1 represents the synthesis of CoTe NRs. In the synthesis process, ascorbic acid covered the surface of the Te nanorods. Co^2+^ ions spread over the carbon shells and react with Te to form CoTe.^40^ CTAB is a cationic surfactant that controls the shape, size and aggregation of nanostructures due to the formation of micelles in the liquid medium. It forms the elongated stable micelles during the addition of CTAB into the water. This can absorb the CoTe nanoparticles and grows as nanorods, and also prevents the further aggregation of the CoTe nanoparticles. The bonding between the CTAB and CoTe nanoparticles enhances the lengthening of the crystals rather than the growth in the axial direction.^41^

### 3.1. Structural and morphological analysis

**Fig. 1:**
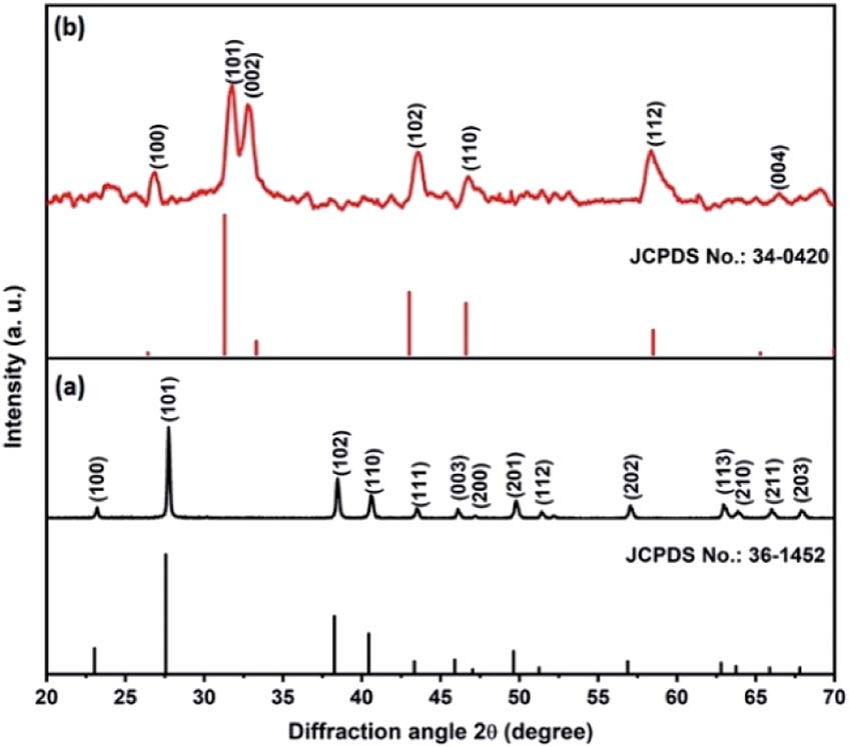
XRD pattern of (a) Te NRs with standard pattern, (b) CoTe NRs with standard pattern.

Fig. 1a shows the XRD pattern of the Te NRs, and the diffraction peaks are well matched with the standard pattern of JCPDS no. 36-1452.^42^ The peaks are well indexed with the hexagonal phase of tellurium with a lattice parameter of *a* = 0.4459 nm and *c* = 0.5854 nm. The diffraction peaks appearing at an angle of 23.2, 27.5, 38.3, 40.4, 43.3, 45.9, 49.6, 51.8, 56.5, 62.8, 63.8 and 67.7° confirmed the corresponding (100), (101), (102), (110), (111), (003), (201), (112), (202), (113), (210) and (203) planes, respectively. The diffraction peaks of the CoTe NRs can be indexed to a hexagonal phase with lattice parameter *a* = 0.3865 nm and *c* = 0.5454 nm, which are coordinated with the standard pattern of JCPDS no. 34-0420 (ref. 35) shown in Fig. 1b. The diffraction peaks appearing at an angle of 26.7, 31.6, 32.9, 43.6, 46.8, 58.3, 64.7 and 69.0° revealed the existence of the (100), (101), (002), (102), (110), (112), (202) and (004) planes, respectively. No additional peaks appeared for both samples, which indicate the high purity of the electrode materials.

**Fig. 2:**
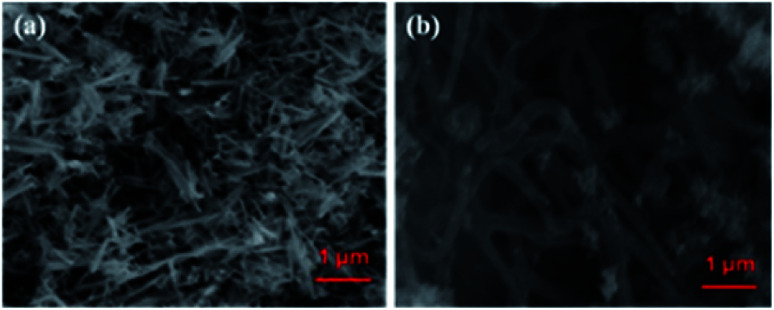
SEM image of (a) CoTe NRs and (b) Te NR.

**Fig. 3:**
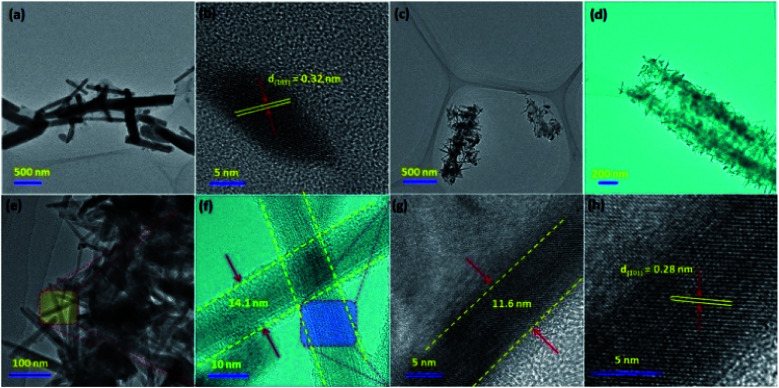
TEM images of Te NRs at (a) low and (b) high magnifications; (c–h) TEM images of CoTe NRs at different magnifications.

The SEM images of CoTe and Te show the formation of uniform nanorod bundles, consisting of nanorods with an average length of up to 1 μm (Fig. 2a and b). The average diameter of the CoTe NRs is less than that of the Te NRs. The samples were further analyzed by TEM analysis. Fig. 3(a and b) shows TEM images of the Te NRs at different magnifications. A lattice-resolved higher magnification TEM image in Fig. 3(b) indicates the interplanar spacing of 0.32 nm, which corresponds to the (101) plane of hexagonal Te and confirms the formation of the Te NRs. Fig. 3(c–e) shows the TEM images of the CoTe NRs at different magnifications. At higher magnification images (Fig. 3f and g), the formation of nanorods are clearly indicated with an average diameter in the range of 11–15 nm, and the surface of each rod was found to be rough. A lattice-resolved higher magnification TEM image in Fig. 3(h) indicates the interplanar spacing of 0.28 nm, which corresponds to the (101) plane of hexagonal CoTe and confirms the formation of CoTe NRs. It was further confirmed that the average diameter of the CoTe NRs is less than that of the Te NRs. These results support the previous analysis (*e.g.*, SEM and XRD analysis).

**Fig. 4:**
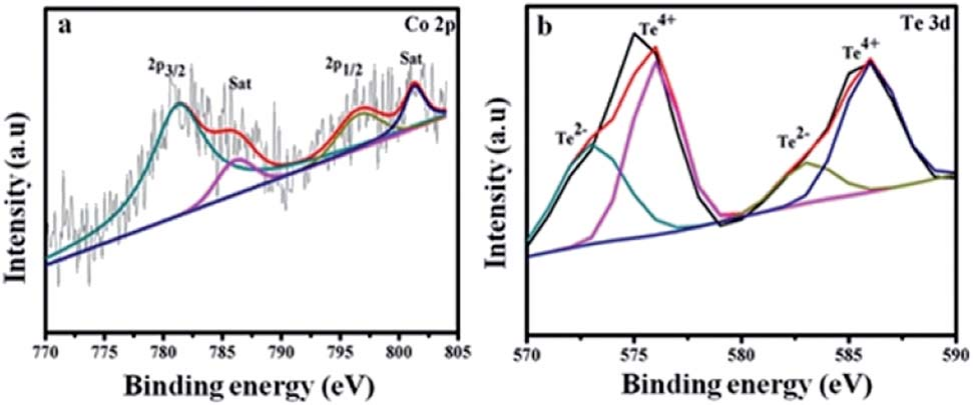
Higher resolution XPS spectra of (a) Co 2p and (b) Te 3d for CoTe NR materials.

Fig. 4a shows Co 2p_3/2_ at 781.2, 786.4 eV and Co 2p_1/2_ at 796.9, 801.4 eV. The energy difference is 15.7 eV for the Co 2p_3/2_ and Co 2p_1/2_ spin–orbit interaction, which reveals the presence of Co^2+^ and Co^3+^ ions.^43^ The Te 3d spectrum in Fig. 4b is divided into four peaks. The satellites at 572 and 583 eV are assigned to Te 3d_5/2_ and Te 3d_3/2_ of the Te^2−^ ions, and the major peaks at 576 and 586 eV arise due to the formation of the Te^4+^ ions.^44^

### 3.2. BET analysis

**Fig. 5:**
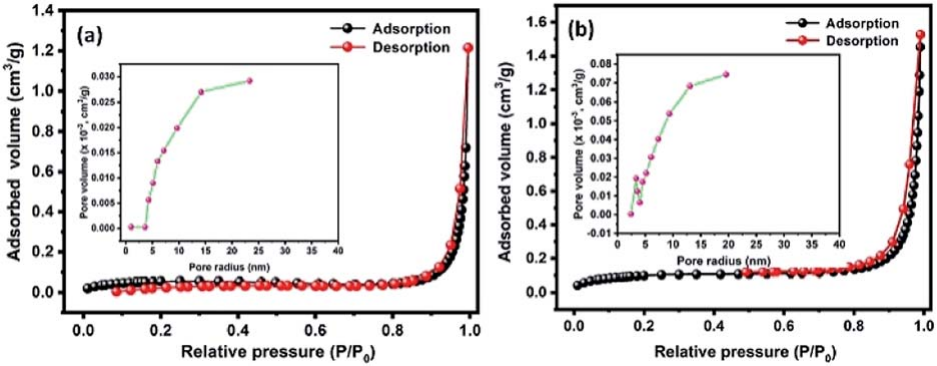
BET N_2_ adsorption–desorption isotherms of the prepared (a) Te NRs and (b) CoTe NRs with corresponding BJH pore size distribution curve (inset a and b).

Fig. 5(a and b) shows the N_2_ adsorption–desorption isotherms of Te and CoTe samples conducted to determine the surface areas of the materials. The product exhibited a type IV H3 characteristic hysteresis behavior, indicating the formation of meso- and microporous structures. The specific surface areas of Te and CoTe were calculated to be 4.8 and 8.4 m^2^ g^−1^, respectively. The Barrett–Joyner–Halenda (BJH) pore size distribution curve indicates the pore radius in the range of 5–14 and 3–12 nm for Te NRs and CoTe NRs (Fig. 5(a and b) (inset)), respectively. The unique meso- and microporous nature with the high specific surface area of the CoTe NRs could improve the mass transport and electron transfer as an electrode in the electrochemical process; this may be an advantage for the excellent electrochemical performance. Therefore, it is estimated that the CoTe NRs can offer higher electrochemical activity than the Te NRs.

### 3.3. Electrochemical performance

**Fig. 6:**
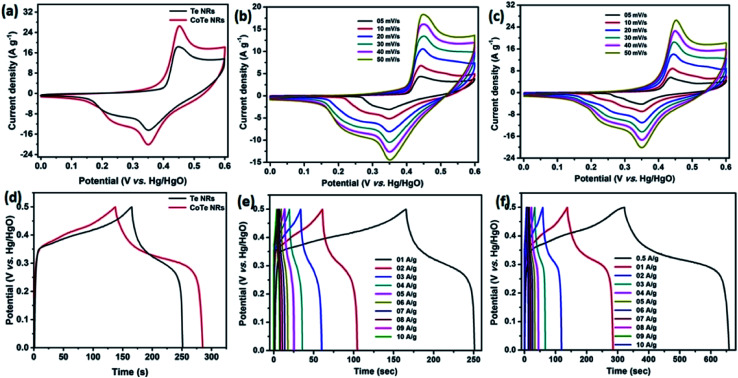
CV profile of the (a) Te and CoTe NRs at a scan rate of 50 mV s^−1^, (b) Te NRs, (c) CoTe NRs at different scan rates; GCD profile of (d) Te and CoTe NRs at 1 A g^−1^ current density, (e) Te NRs and (f) CoTe NRs at different current densities.

Initially, the electrochemical properties of the electrode materials were characterized by CV, GCD and EIS analysis. The CV profile of the Te NR and CoTe NR electrodes at a scan rate of 50 mV s^−1^ showed redox-type voltammograms, which demonstrate the faradaic charge storage mechanism (Fig. 6a). The CV profile of the CoTe NR electrode displayed a wider and high current density than the Te electrode. Fig. 6(b and c) shows the CV profile of the Te and CoTe NR electrode at different scan rates ranging from 5 to 50 mV s^−1^. Both electrode materials have a strong pair of redox peaks during the CV analysis, which resulted from the following surface reversible redox reaction between Co^2+^ and Te for CoTe NRs and Te NRs in the presence of OH^−^ ions, respectively. The possible redox reactions in the Te and CoTe electrodes can be described as follows:^22,45^

8 Te + OH^−^ ⇌ TeOH + e^−^

9 TeOH + OH^−^ ⇌ TeO + H_2_O + e^−^

10 CoTe + OH^−^ ⇌ CoTeOH + e^−^

11 CoTeOH + OH^−^ ⇌ CoTeO + H_2_O + e^−^

While the scan rate increased, the oxidation and reduction peaks shifted to negative and positive potentials, respectively, because the ion diffusion rate had to be limited to satisfy electronic neutralization during the redox process. At a slow scan rate, the OH^−^ ions have enough time to intercalate with the inner and outer surfaces of the electrode. But, in the case of a high scan rate, the OH^−^ ions have less time to intercalate with the surfaces of the electrode.

**Fig. 7:**
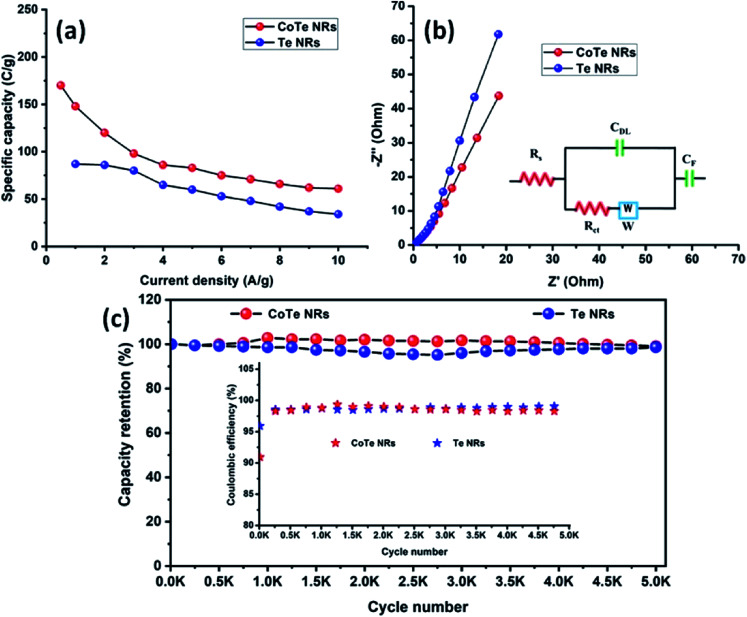
(a) Specific capacity of CoTe NRs and Te NRs as a function of current density; (b) Nyquist plot of CoTe NRs and Te NRs with corresponding equivalent circuit (inset) and (c) specific capacity retention and coulombic efficiency (inset) of CoTe NRs and Te NRs as a function of the cycle number.

The electrode materials were further investigated by GCD analysis at various current densities from 1 to 10 A g^−1^ at the potential range of 0.0 to 0.5 V *vs.* Hg/HgO (20% KOH). Compared to the CV profile, the active potential window was slightly decreased for GCD analysis because of gas evolution at low current density. Fig. 6d shows the GCD profile of the Te and CoTe electrodes. CoTe had a higher discharge time compared to the Te electrode at the current density of 1 A g^−1^. The GCD profile exhibits non-linear charge/discharge profiles that confirm the faradaic nature of the electrode materials, which is in good agreement with the CV analysis. The calculated specific capacities of the Te NRs and CoTe NRs were 87 and 148 C g^−1^, respectively, at 1 A g^−1^ current density. The GCD profile of the Te NR and CoTe NR electrode materials at different current densities are shown in Fig. 6(e and f). The specific capacity of all electrode materials as a function of different current densities is displayed in Fig. 7a. The calculated specific capacities of the Te NRs from the GCD profile are 87, 86, 80, 65, 60, 53, 48, 42, 37, and 34 C g^−1^ at current densities of 1, 2, 3, 4, 5, 6, 7, 8, 9 and 10 A g^−1^, respectively. The calculated specific capacities of the CoTe NRs from the GCD profile are 170, 148, 120, 98, 86, 83, 75, 71, 66, 62, and 61 C g^−1^ at current densities of 0.5, 1, 2, 3, 4, 5, 6, 7, 8, 9 and 10 A g^−1^, respectively. Even at the high current density of 10 A g^−1^, the CoTe NRs and Te NRs delivered a good rate performance of 36 and 39%, respectively. The specific capacitance value decreased while increasing the current density; this may be attributed to the diffusion of ions at different current rates. At a lower current density, the electrolyte ions have a sufficient time to diffuse to the inner pores of the electrode surfaces to attain the maximum utilization of the active surface areas of the electrode materials.^46,47^

At high current densities, the electrolyte ions can access only the surface of the electrode materials. They do not have enough time to intercalate the inner pores of the electrode surfaces to attain the minimum utilization of the active surface areas of the electrode materials. Here, we have compared the specific capacities of some metal chalcogenide-based electrode materials with our synthesized materials. Although the specific capacity of CoTe was slightly lower than that of the reported materials, its capacity retention was much higher even after 5000 cycles. The interfacial resistance of the electrode materials at the electrode and electrolyte interfaces was evaluated using the EIS technique. Fig. 7b reveals the Nyquist plot of the CoTe NR and Te NR electrode materials fitted with an appropriate equivalent circuit model. The equivalent circuit model contained solution resistance (*R*_s_), charge-transfer resistance (*R*_ct_), double layer capacitance (*C*_dl_), Warburg impedance (*W*) or diffusion resistance and faradaic capacitance (*C*_F_). From the fitting analysis, the CoTe NR and Te NR electrodes showed *R*_s_ values of 0.18 and 0.21 Ω, respectively. The *R*_ct_ values were 2.3 and 2.7 Ω for the CoTe NR and Te NR electrodes, respectively. In addition, the CoTe electrode materials showed a vertical line along the *y*-axis in the low frequency region of more than 45°, which implied the ideal capacitive behavior of the electrode materials with less interfacial charge transfer resistance at the electrode/electrolyte interface.

The electrochemical stability is one of the vital properties for a supercapacitor. To assess the cyclic stability of the electrodes, a GCD test was carried out up to 5000 cycles at a high current density of 10 A g^−1^ as shown in Fig. 7c. After 5000 cycles, 98.5% (Te) and 99% (CoTe) of the initial specific capacitance was retained. For all 5000 cycles, the Coulombic efficiency of the electrode materials remained constant (∼98%), which confirms the high reversibility of the charge storage process. This good stability reveals that the highly reversible surface redox reaction took place between the electrolyte and electrode surface. To this end, the CoTe NR electrode shows higher electrochemical activity than the Te NR electrode. For the full cell analysis, CoTe NRs was used as a positive electrode and OPAA was used as the negative electrode for further studies.

### 3.4. Hybrid asymmetric supercapacitor

**Fig. 8:**
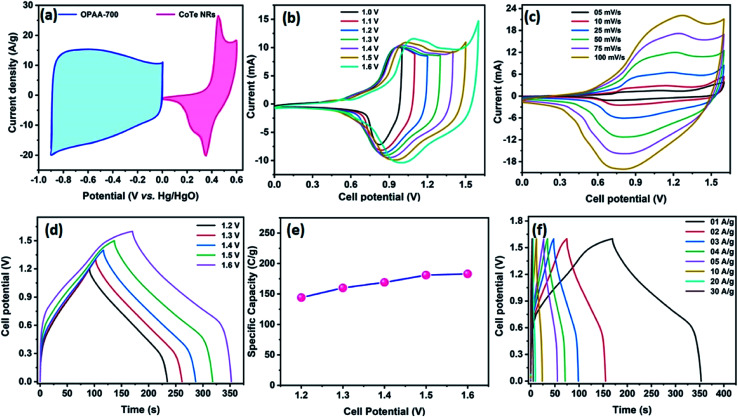
(a) CV profile of OPAA and CoTe electrodes from the half-cell (three electrode configuration) analysis at a scan rate of 50 mV s; CV profile of CoTe‖OPAA ACS (b) at the scan rate of 50 mV s^−1^ with different cell potentials and (c) at different scan rates with a cell potential of 1.6 V. GCD profile of the fabricated ASC at (d) different cell potentials at 1 A g^−1^ current density and (e) specific capacity obtained as a function of different cell potentials, (f) at different current densities with the cell potential of 1.6 V as a cell voltage.

Fig. 8a represents the CV profile of OPAA and CoTe electrodes occupying different potential windows of −1.0 to 0 V and 0 to 0.6 V at the scan rate of 50 mV s^−1^, respectively. The CV profiles of the hybrid ASC (CoTe‖OPAA) at different cell potentials from 1 to 1.6 V at the scan rate 50 mV s^−1^ are shown in Fig. 8b. The device remained stable with increasing cell potential up to 1.6 V. The set of redox peaks with a wider CV profile indicates the existence of faradaic and EDLC nature properties. At the cell voltage of 1.6 V, the CV curves of the CoTe‖OPAA hybrid ASC at different scan rates from 5 to 100 mV s^−1^ are given in Fig. 8c. While the scan rate increased, the oxidation and reduction peaks shifted to negative and positive potentials because the ion diffusion rate had to be limited to satisfy electronic neutralization during the redox process. At a slow scan rate, the OH^−^ ions have enough time to intercalate with the inner and outer surfaces of the electrode. However, in the case of a high scan rate, the OH^−^ ions have less time to intercalate with the surfaces of the electrode.

**Fig. 9:**
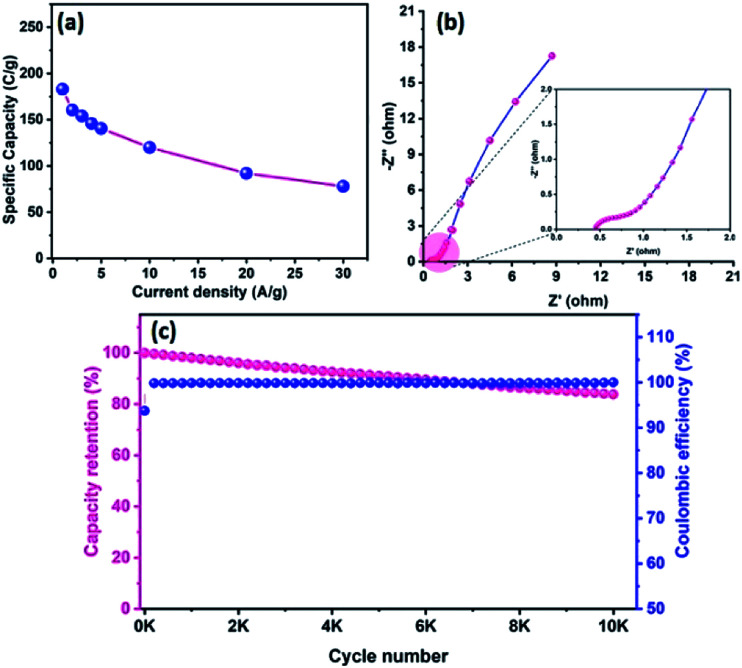
(a) Specific capacity curve of fabricated ACS as a function of different current densities, (b) Nyquist plot of CoTe NRs‖OPAA ASC with the magnified view (inset), and (c) the capacity retention and coulombic efficiency of fabricated ASC as a function of cycle numbers.

Fig. 8d reveals the GCD curves of the hybrid ASC device performed at a current density of 1 A g^−1^ in different cell potentials (1.2 to 1.6 V). From the discharge profile, the calculated specific capacity of the hybrid ASC cell was 144, 160, 169, 181 and 183 C g^−1^ at cell potentials of 1.2, 1.3, 1.4, 1.5 and 1.6 V, respectively. The specific capacity as a function of the cell potential is shown in Fig. 8e, where the capacity value increased with increasing cell potential. Even at 1.6 V cell potential, the CD profile shows a peak-like charge–discharge profile without any gas evolution. Fig. 8f demonstrates the GCD profile of the hybrid ASC cell with a cell potential of 1.6 V at different current densities. The calculated specific capacity of the hybrid ASC cell was 183, 160.4, 154, 146, 140.5, 120, 92 and 78 C g^−1^ at the current density values of 1, 2, 3, 4, 5, 10, 20 and 30 A g^−1^, respectively. When the current density was increased to 30 A g^−1^, the hybrid ASC cell retained 42.6% of the initial specific capacity (Fig. 9a). Fig. 9b Illustrates the Nyquist plot of the fabricated hybrid ASC cell, which exhibited *R*_s_ and *R*_ct_ values of ∼0.4 and ∼0.6 Ω, respectively. From the EIS studies, it was confirmed that the fabricated hybrid ASC cell had excellent electrical contact with minimum ionic resistance.

For viable applications, a supercapacitor should have high electrochemical stability. In order to find the electrochemical stability of the fabricated hybrid ASC cell, cycling studies were carried out for 10 000 cycles at the high current density of 30 A g^−1^. Fig. 9c shows the capacity retention and coulombic efficiency of the hybrid ASC cell as a function of the cycle number. The fabricated hybrid ASC cell delivered an excellent electrochemical stability of 85% of the initial specific capacity after 10 000 charge–discharge cycles with a coulombic efficiency of 99.9%. The fabricated hybrid ASC cell with CoTe NRs and OPAA electrode materials as the positive and negative electrodes shows good discharge reversibility and outstanding cycle stability, respectively. These electrochemical stability values are higher than the recently reported materials, such as CoTe‖AC (90% for 5000 cycles),^21^ CoTe‖AC (80% for 3000 cycles),^22^ NiTe (81% for 3000 cycles),^25^ 1T-MoTe_2_ (87% for 5000 cycles).^26^

**Fig. 10:**
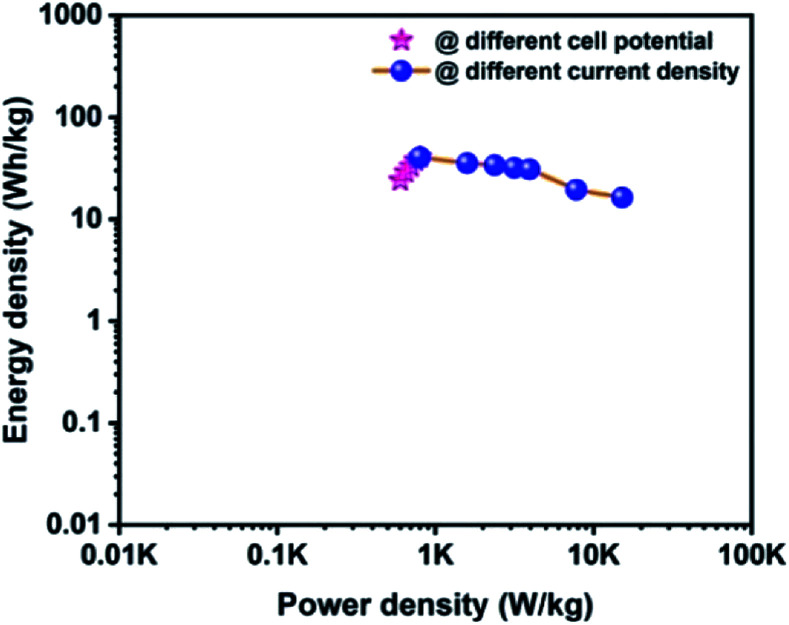
Ragone plot for the fabricated ACS as a function of different cell potentials and different current densities using CoTe NRs and OPAA as the positive and negative electrodes, respectively.

The Ragone plot is a typical tool to evaluate the supercapacitor device with other types of energy storage devices in terms of their energy and power density values. Fig. 10 displays the Ragone plot of the fabricated CoTe‖OPAA hybrid ASC device. The fabricated hybrid ASC device exhibited high energy density values of 24, 28.9, 32.9, 37.7 and 40.7 W h kg^−1^ with cell potential values of 1.2, 1.3, 1.4, 1.5 and 1.6 V, respectively. Notably, the energy density of the device increased with increasing cell potential. Similarly, the power density also increased to 600, 650, 701, 750 and 800 W kg^−1^ with increasing cell potential at 1.2, 1.3, 1.4, 1.5 and 1.6 V, respectively (Fig. 10).

The high energy and power density values of the fabricated hybrid ASC at a current density of 1 A g^−1^ at the cell potential of 1.6 V were 40.7 W h kg^−1^ and 800 W kg^−1^, respectively. When the current density increased to 30 A g^−1^, the fabricated hybrid ASC device delivered a high-power density of 22.5 kW kg^−1^ and retained an energy density of 16.3 W h kg^−1^. The above observed high energy and power densities were significantly higher than the reported values for the metal oxide-based electrode materials in ASC using an aqueous electrolyte. For instance, Ye *et al.* synthesized CoTe‖AC ASC that delivered an energy density of 67 W h kg^−1^ for the pristine electrolyte.^22^ Quan *et al.* fabricated a CuS-CNTs@NF‖AC ASC showing the specific capacitance of 164 F g^−1^ at a current density of 0.5 A g^−1^ with an energy density of 22.14 W h kg^−1^.^48^ The assembled Ni_3_N‖PRPC-1K ASC possessing a high energy and power density of 30.9 W h kg^−1^ and 412 W kg^−1^, respectively, have been reported by Peng *et al.*^49^ Wang *et al.* constructed a NiCo LDH‖AC ASC displaying a specific capacitance of 58 F g^−1^ at the current density of 1 A g^−1^ with an energy and power density of 18 W h kg^−1^ and 750 W kg^−1^, respectively.^50^ The remarkable electrochemical performance of the fabricated hybrid ASC can be attributed to the good combination of the faradaic-based CoTe NRs and EDLC-based high surface area activated carbon (OPAA) CoS nanoparticles and activated carbon electrode. In addition, this methodology could be readily extended to the preparation of other metal tellurides.

## 4. Conclusions

In this work, a simple hydrothermal method has been used for the synthesis of Te NRs and CoTe NRs. The XRD pattern revealed the formation of hexagonal CoTe NRs, and the compositional analysis confirmed the presence of Co and Te. The prepared materials showed a significant enhancement in the electrochemical performance when they were used as electrode materials in a supercapacitor. The CoTe NR electrode shows a high specific capacity of 170 C g^−1^ at a current density of 0.5 A g^−1^ and excellent life cycle (99%). The fabricated CoTe‖OPAA ASC achieved a specific capacity of 183 C g^−1^ with a high-energy density of 40.7 W h kg^−1^ and power density of 800 W kg^−1^ at the current density of 1 A g^−1^. The device delivered a high power density of 22.5 kW kg^−1^ with an energy density of 16.3 W h kg^−1^ at a high current density of 30 A g^−1^. The fabricated hybrid ASC cell delivered an excellent electrochemical stability of 85% of the initial specific capacity even after 10 000 charge–discharge cycles with a coulombic efficiency of 99.9%. The prepared materials have shown a promising results as an electrode for supercapacitor applications. The proposed simple hydrothermal method could be scaled up for a large-scale preparation of CoTe and other Te-based materials for their viable applications in energy storage. Furthermore, these materials have a great potential in other applications such as lithium-ion batteries, catalysts and data storage devices.

## Conflicts of interest

There are no conflicts to declare.

## Supplementary Material
